# Clinical and Experimental Use of Orthoform

**Published:** 1900-07

**Authors:** August Luxenburger

**Affiliations:** Assistant to the Surgical Polyclinic of the University of Munich


### Selection.

### CLINICAL AND EXPERIMENTAL USE OF ORTHOFORM.

By DR. AUGUST LUXENBURGER,
Assistant to the Surgical Polyclinic of the University of Munich.

Reprint from the *Münchener Medizinische Wochenschrift,* Nos. 2 and 3, 1900.
[*Translated for the* BUFFALO MEDICAL JOURNAL.]

SINCE the first publication, over a year ago, of the reports of Prof. Klaussner and Dr. Kallenberger upon the favorable results obtained with the local anesthetic orthoform, this preparation has been extensively used upon the ample material offered in the surgical ward of the Polyclinic of the University of Munich. It is, therefore, opportune, after these exhaustive experiments and the collective reports of numerous other authors, to give a conclusive opinion upon this new preparation, which will justify the adoption of orthoform as a remedial agent of the physician and particularly of the surgeon. The surgeon, above all others, demands of a new chemical agent, that, next to its primary object—in this case local anesthesia—the remedy should be inocuous, *i.e.*, should neither retard the process of healing or in any way interfere with the general organism.

As a prime condition, therefore, orthoform as delivered from the laboratory must not contain infectious germs. Repeated experiments with agar, gelatine and bouillon have shown that orthoform fully answers this prime condition—sterility. Lichwitz and Sabrazes expressed the same favorable opinion. After their subsequent experiments with this remedy they came to the conclusion that orthoform possessed a moderate antiseptic action.

Its strongly antiputrifactive quality in contact with albumin was pointed out by Kallenberger and corroborated by Mosse, who also mentioned that it was possible to prevent the ammonia fermentation of urine by the addition of orthoform. Of this fact I have been able to convince myself also. An addition of 0.5 per cent ($\frac{1}{2}$ per cent.) of orthoform to urine will not only prevent the ammonia fermentation for weeks, but fermentation already begun may be resisted by this quantity and fetid smelling urine rendered almost odorless. Alcoholic fermentation produced by yeast in saccharin urine is also considerably retarded by orthoform. To further determine the antiseptic properties of orthoform, which were classified by Liebwitz and Sabrazes as "moderate," I instituted a number of experiments with cultures of various microörganisms, especially those promoting suppuration, with the addition of orthoform, using the customary media for propagation. The results are shown in the following experiments.

Orthoform was strewn upon fresh coatings of agar cultures of bac. pyocyaneus, staphylococcus citr., and streptococcus, and the same cultures, for comparison, treated with lycopodium and iodoform. Transplantations showed that under lycopodium and iodoform the cultures flourished without hindrance, while those under orthoform remained sterile. These experiments made with orthoform conclusively proved that it possessed the property of arresting the development of bacteria, and it now remained to follow this up and establish the quantitative strength of this property. This was comparatively easy as orthoform is soluble up to 1 per cent. in agar, gelatine and blood serum, showing a light, yellowish brown discoloration due to the slightly alkaline reaction of the former.

TABLE NO. I.

| Per cent. of Orthoform. | 1.0%. | 0.5%. | 0.32%. | 0.25%. | 0.17%. | 0.125%. | 0.1%. | 0.05%. |
|---|---|---|---|---|---|---|---|---|
| Bac. pyocyan ... | No colonies. | No colonies. | No colonies. | Few colonies, sparse. | Light covering. | Thick covering. | Thick extensive covering, no coloring. | Very strong growth, some odor, no coloring. |
| Staphyl. citreus | No colonies. | No colonies. | No colonies. | No colonies. | Isolated colonies. | Moderate light yellow covering. | Thick yellow covering. | Thick yellow covering. |
| Streptococcus... | No colonies. | No colonies. | No colonies. | No colonies. | Isolated colonies. | Moderate covering. | Moderate covering. | Plentiful covering. |

See results obtained in the following experiments made with orthoform gelatine of various percentages: Ten c.cm. each of variously prepared gelatines were inoculated respectively with an iota of bouillon culture of bac. pyocyaneus, staphylococcus aurens, etc., and after being thoroughly incorporated into this medium were poured into equal sized petri-discs. After standing two days at a temperature of 22° C. the results shown in the following table were obtained:

TABLE NO. II.

| Per cent. of Orthoform. | 1.0%. | 0.5%. | 0.32%. | 0.25%. | 0.17%. | 0.125%. |
|---|---|---|---|---|---|---|
| Bac. pyocyan........... | No colonies. | No colonies. | No colonies. | No colonies. | Average 10 colonies in the field of vision. | Countless colonies, no coloring. |
| Staphyl. citreus....... | No colonies. | No colonies. | No colonies. | No colonies. | Average 4 colonies in the field of vision. | Average 9 colonies in the field of vision. |
| Streptococcus ......... | No colonies. | No colonies. | No colonies. | No colonies. | Average 13 colonies in the field of vision. | Average 35 colonies in the field of vision. |

The plates which contained the c.5 per cent.—0.32 per cent. and 0.25 per cent. (respectively ½ per cent., ⅓ per cent., and ¼ per cent.) of orthoform gelatine, and which had not shown a trace of bacteria growth, were now gradually melted at a degree of 37° C., and the percentage of orthoform reduced to ⅓ of the former strength by the addition to each of twice the volume of gelatine. After forty-eight hours a more or less luxuriant growth of bacteria was noticed, which proved that 0.5 per cent. 0.32 per cent. and 0.25 per cent. was not sufficient to destroy the germs although it had checked their growth.

Experiments with blood serum were made with orthoform and gave the following results :

Table No. III.

| Per cent. of Orthoform. | 1%. | ½%. | ⅓%. | ¼%. | 1-5%. | 1-10%. | 1-15%. | 1-20%. |
|---|---|---|---|---|---|---|---|---|
| Bac. pyocyan. | No colonies. | No colonies. | No colonies. | No colonies. | Colonies very sparse. | Few colonies. | Very many colonies. | Thick covering, no coloring. |
| Staphyl. citr.. | No colonies. | No colonies. | No colonies. | No colonies. | No colonies. | Few weakly colonies. | Abundant pale yellow covering. | |
| Streptococcus | No colonies. | No colonies. | No colonies. | No colonies. | No colonies. | Moderate covering. | Thick covering. | |

These experiments were further backed up by another, of a similar nature, made by Lichwitz and Sabrazes. Bouillon cultures of pyocyaneus and staphylococcus albus, which were several days old, were thoroughly mixed with orthoform, placed in an incubator and frequently shaken up. Transplantations made from them after three or four days proved that the germs of the former had been entirely killed.

More importance should perhaps be attached to another circumstance. In the accompanying tables it is frequently shown that under the influence or orthoform, even of the lowest per cent., bac. pyocyaneus develop scarcely any color, and produce but little odor, and that staphylococcus citrus grew only pale yellow. Futhermore, it was easily seen that transplantations from orthoform cultures were much slower to darken bouillon, apparently, as if they had to recuperate; in short, bacteria which had been raised under the influence of orthoform, seemed to have lost much of their vitality. This made room for the hope of eventually reducing their virulence in like manner. This question was solved by the following experiment. Three rabbits were inoculated each with ½c. cm. of a virulent streptococcus culture, but with this difference:

A was inoculated with a culture that had been kept for ten hours thoroughly mixed with orthoform: B with culture under influence of orthoform for five minutes and C with pure culture. Rabbit C died of sepsis after 1½ days; cocci were found in blood and organs. B developed a large abscess and died in three days; post mortem showing like conditions as C. A remained well, showing at the point of injection only a slight, rapidly disappearing swelling. In these experiments I believe to have sufficiently demonstrated the not inconsiderable antiseptic value of orthoform.

As no germs are found in orthoform when it leaves the laboratory, and all germs that might accidentally find their way into it by exposure, are either entirely annihilated or lose their virulence by contact with it, the remedy may always be used without further sterilisation. As orthoform cannot be classed as an antiseptic, it is, therefore, necessary to combine with its use some antiseptic of known quality, as it is also necessary for pertinent reasons to sometimes combine with it some substances that will assist granulation and others that will retard secretion. It was, therefore, desirable to know what effect orthoform would have upon these various chemicals when in continued contact with them, and if applied simultaneously there might not result some unknown chemical compound that could eventually prove dangerous to the wound. In this direction the author has made a number of experiments which prove beyond question that among the commonly used disinfectants formalin, nitrate of silver and permanganate of potassium solutions should not be used in conjunction with orthoform. Corrosive sublimate does not lose its efficiency by the addition of orthoform. Turpentine, tincture of iodin and solution of copper sulphate are indifferent to its action. A combination of ichthyol offers advantages inasmuch as glycerin or collodion may be used as a vehicle. Other good combinations with orthoform are: Iodoform, thioiodoform, dermatol, zinc oxide, euorphen, aristol, calomel and salicylic acid. Bismuth subnitrate, mixed with orthoform assumes a chocolate brown color, and it is now advisable to use them in combination as a powder. Carbolic acid in 3 per cent to 5 per cent. solutions is not affected, neither are lysol or kreosol solutions. Lead water, borates and acetate of alumina do not decompose orthoform.

In the following numerous cases orthoform was used sometimes alone, but mostly in combination with some of the chemicals above cited: 35 cases of more or less infected bruised finger tips, with or without fracture of the phalanges, broken joint or torn tendons.

Treatment: Dusting with pure orthoform; bandaging with moist boric acid or acetate alumnia gauze once daily for two or three days.

Twenty-two cases of severe cuts, especially upon the hands and the lower limbs: Treatment: Dusting with orthoform for three or four days, and bandaging with iodoform gauze, or, where infection was apparent or suspected with acetate alumina gauze.

Twenty cases of infected incised wounds, mostly with lymphangitis. Treatment: Three or four days' daily application of orthoform powder, covered with moist bandages of lead water or solution of corrosive sublimate. Clinical observations did not reveal any weakening of the corrosive sublimate action in the presence of orthoform.

Twenty-eight cases of abrasions of the skin, particularly on the vola manus and on the shins. Treated with 5 per cent. to 10 per cent. orthoform-dermatol salve where no infection existed.

Thirty-one cases of burns, of the second degree, with benzin, alcohol, etc., including scalds from steam and burning with acids—mostly of the hands face and forearms. These cases which came under treatment immediately after the accident, with possibly no infection, were treated after opening of the blisters, with a 10 per cent orthoform-dermatol starch powder, and later with a 10 per cent. orthoform-dermatol vaselin salve, if the secretions remained within moderate bounds. Signs of infection were followed up with applications of lead water, and the surface of the wounds dusted over with orthoform.

Eight cases of running ulcers, mostly on the lower limbs, some reaching to the bone. Daily application of orthoform for eight to twelve days in combination with moist corrosive sublimate or iodoform gauze or mercurial ointment. Dirty looking surfaces of ulcers were often successfully cleaned by the application of three parts of calomel and one part orthoform and bandaging with chloride of sodium solution.

Five cases of tubercular ulcers, two on the neck and three in the mouth required many weeks of treatment with equal parts of orthoform and iodoform.

Five cases of traumatic ulcers on the foot, mostly acccompanied by lymphangitis; treated by dusting with orthoform and bandages of acetate of alumina.

Three cases of decubitus—ulcers on the back of the size of a hand. One very greasy, coated and fetid ulcer; the odor was destroyed by the use of orthoform alone. In this, as in other cases the ulcers were cleansed by powdering with orthoform; application of lead water bandages without gutta percha; finally, orthoform-dermatol powder or salve. All healed.

Four cases of trophic ulcers on the volar side of the great toe, tabic, 2; diabetic, 1, arteriosclerotic, 1. Application of orthoform and iodoform, or orthoform and glutol, or orthoform-ichthyol salve. Where much swelling surrounded the ulcers orthoform and salicylic acid—equal parts—were used. Two healed; others improved.

Fifteen cases of varicose ulcers on the lower limbs; mostly highly painful, so-called erethic ulcers of various sizes; some with symptoms of moderate infection, secreting freely; some with atonic, swollen edges and serous secretion, and poorly granulating ground. The troublesome erethic ulcers were soon relieved by powdering with a mixture of orthoform-dermatol and talcum powder or salve over which a dry gauze was placed, held by a centripetal elastic bandage. When much pain prevailed, with obstinate healing, strict confinement to the bed was ordered. During the day oft repeated applications of Burow's solution, and for the night orthoform powder and bandaging with lead water. Foot ulcers which were much inflamed were treated twice daily with moist applications of acetate of alumina and once daily powdered with orthoform and later sewed up with a 5 per cent. gauze compress of orthoform clay. This was of great advantage where there was much secretion. In atonic ulcers orthoform was only used to allay the pain attending the necessary application of a cautery. Applications of three parts of calomel and one part orthoform, over which wet compresses of chlorid of sodium are placed, were borne without pain. A cautery of a 2 per cent. to 5 per cent. solution of chlorid of zinc, or tincture of iodine, may also be applied without pain if orthoform anesthesia has been resorted to twenty minutes before applying the cautery. To prevent crusting, and also to facilitate the removal of remnants of powder, it is advisable to cover the ulcer with a coarse gauze before applying the orthoform powder. In this manner all surplus powder may be easily removed. Finally, in cases where moist applications are indicated for a number of days, the parts surrounding the ulcer are covered with a thick paste of oxide of zinc. Of the fifteen cases cited, ten healed with healthy scars, without operative process; four were still under treatment with prospective early recovery, and one case apparently incurable on account of general decrepitude. Among the patients are some who have used the orthoform treatment for six weeks.

In two cases of chancroid, orthoform was applied twenty minutes before applying a cautery of sulphate of copper, with the effect of reducing the enormous painfulness to a minimum.

Of five carcinomatous ulcers, one on the tongue, showed the most pronounced orthoform reaction. The anesthetised patient was able to eat comfortably once more, and to have quieted the rebellious trigemini for three to five hours. The same happy results were obtained in the treatment of an ulcerating carcinoma of the penis. Here also the fetid odor rapidly disappeared upon dusting with orthoform. One patient with rectal and anal carcinoma experienced much relief from 10 per cent. orthoform suppositories. Another suffering with tonsillar carcinoma was relieved only as far as deglution was concerned—the pains radiating to the ear continued on. No disturbance of the digestive functions followed the orthoform unavoidably swallowed. In three cases of anal fissure the introduction of cocoa butter suppositories, containing 10 per cent. orthoform allayed the troublesome burning that followed stool. Powdering with orthoform permitted painless cauterisation with argent. nitr., which followed twenty minutes later.

In five cases of ingrown nails pain ceased and the granulations disappeared in a few days upon the application of equal parts of orthoform and salicylic acid.

In four painful fistulæ—1 anal, 1 urethral and 2 tubercular—the latter on the neck, cocoa butter points or pencils containing 5 per cent. orthoform were used with excellent results.

Three ulcers following frost-bite were rapidly healed with an ichthyol-orthoform salve of the following formulæ: ichthyol, 3.0; orthoform, 2.0 and vaselin, 30.0

In the cases so far cited, 198, the object of the application of orthoform, anesthesia was fully realised and anesthesia usually set in within three to five minutes, rarely later than eight minutes, accompanied only by a slight burning sensation, provided, however, that lesion of the skin would allow the remedy to come into direct contact with the nerve terminals. Anesthesia can be kept up for a long time by the deposit of a larger quantity of orthoform, as small quantities come into action from time to time until all is used up. Where orthoform is less adherent, as for instance, in tonsillar carcinoma, and, therefore, the deposit of a quantity impossible, also on account of localisation, or in cases where abundant secretion washes the powder away, the intensity and duration of the anesthesia is necessarily lessened. This can be compensated for by oft repeated applications. The average duration of the anesthesia was ten hours, at one time it lasted two days. Anesthesia lasting seven days was observed by Hecker after insufflation of orthoform in a case of laryngeal ulcer. The intensity of the anesthesia may attain such a degree that curetting may be undertaken upon granulated surfaces preparatory to skin grafting. The manner of application for this purpose is as follows: Two or three hours before curetting is intended, the granulations are freely powdered with orthoform over which a gauze saturated in boric acid and alcohol is placed, and over this a Billroth battist. The good services rendered by orthoform in the hands of the surgeon in accidental surgery gave rise to the idea of using orthoform to allay the afterpains following operations. In this direction others have also made use of orthoform and found it very serviceable. Blondel recommends tamponing with orthoform after curetting of the uterus. Gompers has used it after operating on the nose and ear. Dreyfus used orthoform after operating for phimosis. If it should be found that orthoform will give the patient the much needed rest, after an operation, its use would be much to be preferred to morphin injections, as orthoform does not seem to affect the general condition.

The following cases demonstrate that by the application of orthoform after pains are entirely prevented or reduced to a minimum in many instances, so that it is seldom necessary to resort to morphin injection.

In eight cases of transplantation of skin, an application of a 10 per cent. orthoform salve prevented the burning pain usually following the excision of the skin. After tamponing with orthoform in incisions of five abscesses in various regions, and two ulcerating bursities of the olecranon the general condition remained unchanged.

Sixteen panarites, five paronchial and six phlegmonic, gave no further trouble after incision and tamponing with orthoform gauze.

Six furuncles and two carbuncles were rendered painless by insufflation of orthoform and bandaging with acetate of alumina.

In five cases of curetting of cheesy, tuberculous glands of the neck, and actinomycoses of the skin of the throat, the patients obtained relief from orthoform and fell into a sound sleep. The same may be said of three cases of curetting of inguinal buboes.

In nine cases of curetting of tubercular bone craters, and the use of orthoform and iodoform tampons, the afterpains that followed were only very slight.

In seven thermocauteries—for angioma, 1; lupus, 2; nevus, 2; anal-fistulæ, 2; orthoform-dermatol salve or gauze did very good service.

In six cases of dental narcosis, insufflation of orthoform prevented not only the appearance of all afterpains, but also all fetid odor.

In operating on three ingrown nails and removal of the nail, good results were obtained with orthoform gauze. These last three operations formed the initiative to the use of orthoform after asepsic operations.

While the need for a remedy to allay afterpains was not so great in these cases as in the preceding cases still operations on the periosteum and tendons, which are generally followed by intense afterpains for one or two days, left room for a desirable and safe anesthetic. As possible disadvantages that might result from the use of orthoform, besides the one already disposed of—infection of the wound—the only other one to be feared was a possible hindrance of primary contact. That this was not the case was satisfactorily demonstrated by various experiments upon animals. The thigh muscles of a rabbit were crushed by means of a clamp, orthoform freely applied and then sewn up. Perfect healing resulted in a few days without extra secretion. In another experiment the sinews were cut through, sewn together and freely powdered with orthoform. These were thoroughly grown together in sixteen days. The radius in two other animals were sawed through, treated with orthoform and were found to knit in three weeks.

As subjects for clinical experiment, the numerous cases of atheroma of the face and back were preferably selected. As these atheroma often occurred in a multiple form and patients often had a number of them removed at one sitting this gave a chance for comparative treatment. A variation in the time of healing of the excisions treated with or without orthoform could not be noted, but one not very serious symptom appeared. Wounds already dry would sometimes develop, for a short time, parenchymatous bleeding when the orthoform had begun to act. The wound then shows at first a gray, and afterward a blackish-brown discoloration. The supposition that this meant a change of the coloring matter of the blood into methemoglobin was not verified, as a spectroscopic examination of defibrinated blood, mixed with orthoform, after some time plainly shows the line of reduced hemoglobin, or, shaken up with air, the two characteristic lines of oxyhemoglobin. The brown color of the blood is, therefore, evidently the result of the discoloration of the orthoform, which has been noticed in powder crusts, on wounds, after two or three days.

The process of healing in 25 atheromæ, 2 lipomæ and 4 operations on glands further demonstrated that the presence of orthoform prevented infection of the wounds. In the following minor operations there was no more suppuration from the use of orthoform: 4 extirpations of buboes, 4 excisions of lupus and carcinoma, 9 finger amputations, 5 incisions in fingers to remove foreign substances, 2 operations for phimosis and 5 sutures of sinews. The wounds were dusted with orthoform, and, in consequence, the dreaded first night after the operation was made much more bearable to the patient, so that here also the use of the remedy was justified. The hope that hereafter the use of morphin injections, even after the largest surgical operations, could be entirely dispensed with will hardly be realised as the necessary doses of orthoform for this purpose would be too large. Although our experience so far has taught us that orthoform is non-poisonous, it is only so on account of its slight solubility. Dogs sustain 3-6 grms. of the substance internally; 3 grms. in the skin. Rabbits who had 1-1½ grms. distributed in a pocket of the skin showed no toxic symptoms. A patient of Klaussner received weekly 60 grms. applied to a carcinoma, without disturbance. Neumeyer says he saw no special symptoms following the medication of 3-4 grms. per day. In these cases, however, no very large absorbent surfaces came into play, as in the amputation of an arm or leg when innumerable exposed veins and lymphatics would absorb large quantities within a short time, even of corpuscular elements. That it is advisable, however, to exercise some care in the use of orthoform was demonstrated by experiments made by Soulier and Guinard. They report fatal results from a quantity approximating 0.5 gm. to each kilo of the weight of a dog when introduced into the peritoneal cavity. Injected into the veins of a rabbit 0.012 gm. per kilo of the weight of the rabbit was sufficient to cause death. These are quite considerable quantities and from these doses we may deduct that a man could stand several grammes of orthoform very well, introduced into a wound. Some of the cases of burns cited above had 2 gms. dusted upon the very absorbent wounds. In the following cases, in which the wounds, some of them quite extensive, were treated with 1 grm. orthoform, no symptoms of intoxication could be discovered in spite of the ready absorption of the remedy. After two hours the violet orthoform reaction could be determined in the urine, with chloride of iron test.

In three cases of necrotomy of the tibia for osteomyelitis the bone plate was thoroughly smeared over with orthoform, and the periosteum and the edges of the skin dusted with orthoform. One was tamponed with iodoform gauze, and two rapidly healed with formation of the Schede blood scab. The usual severe afterpains were reduced to a minimum. Also three radical operations for hernia on two patients. In the double operation only the wound of the right side was treated with orthoform-boric acid powder in the proportion of 4 to 1. Healing resulted by first intention in ten days. Secretion of both sides was of a moderate quantity. In the first few days after the operation patient experienced no inconvenience from the right, but a burning and sharp pain in the left side.

One man with incision in the bladder complained only of inconvenience from the drainage catheter.

In three amputations of the leg below the knee—one for arteriosclerotic gangrene and two for gangrene following frozen limbs—the orthoform-boric acid powder, above mentioned, was applied particularly to the severed nerves, to the periosteum and upon the fascia and segments of the skin. The usually sharp burning pains were considerably modified.

In one case of tracheotomy nothing of moment occurred.

As to the usefulness of orthoform and the advisability of its application in capital (major) operations it is rather too soon to give a conclusive opinion, on account of the few cases of major operations treated with orthoform. The cases so far treated would certainly encourage and justify its further use.

Czerny and Trunececk frequently mention the use of orthoform to modify the otherwise very painful injection of arsenic. Pouchet recommends calomel-orthoform injections.

By the great number of iodoform-glycerin injections given in the clinic it was demonstrated in many cases the addition of 5 per cent. of orthoform proved very useful and at the same time harmless. The eminent good services rendered by orthoform injection in painful affections of the bladder deserve special mention.

Three cases of stone in the bladder—one with slight and two with pronounced cystitis and frequent bloody discharge—were treated daily at night with 1 gm. orthoform suspended in a small quantity of physiological sodium chloride solution, and injected after washing out with boric acid. The frequency of the micturitions was thereby reduced from 16 to 3 at night, and the accompanying tenesmus reduced in violence. The irritation of the muscles of the bladder, which at first reduced the capacity of the bladder to 50 c.cm. was so much allayed that in a few days the capacity was increased to 100-200 c.cm. The patient was also enabled to move about more freely without fear of spasms of pain produced by the moving calculi. One case of tubercular cystitis proved equally satisfactory, while two cases of gonorrhoic cystitis did not seem to react to the orthoform treatment. Complete success was obtained in the treatment of two cases of prostatitis with purulent cystitis. Besides the very remarkable subjective improvement, the rapid improvement in the condition of the urine was surprising. The urine soon lost its fetid odor—by virtue of the quality of orthoform already mentioned—to stop the ammonia fermentation. These cases reacted much quicker with orthoform than, under the usual treatment with boric and salicylic acid irrigation and internal medication with urotropin. The same may be said of a case of severe cystitis complicated with traumatic stricture of the urethra and uric fistula of the perineum. In this, as in another similar case, a drainage catheter was introduced after excision of the stricture had been performed, and the catheter kept there for some time. As a lubricator for this catheter a 10 per cent. orthoform vaselin was used. Another very thin elastic catheter was also frequently introduced beside the durable catheter, and an injection of orthoform oil—orthoform, 1.0; olive oil, 20.0—made into the neck of the bladder and pars prostatica. To this manipulation is ascribed the fact that in one case the durable catheter was comfortably borne for three weeks. One patient with carcinoma of the bladder, showed marked improvement of the subjective symptoms and of the condition of the urine. The same patient received orthoform treatment for several weeks without apparent disadvantage.

The injections generally being given at night— 1-1½ gm. to 2 gm. orthoform—had a most beneficial influence upon the painful tenesmus, the tension of the bladder, number of micturitions and condition of the urine; in all cases except the two of gonorrhoic cystites above cited. The failure in these cases of a reaction upon the subjective symptoms was due, as in most cases of affections of the skin and mucous membrane, to the probable absence of ulcerative process. In two cases, one of tubercular, the other of prostatic, purulent cystitis, the cystoscope revealed ulcers with raised edges. It is therefore, rational to assume, from the marked influence upon the subjective symptoms, also in the other cases (especially where calculi was present) the presence of suppurative process, and to claim for orthoform the same diagnostic value in affections of the bladder as it is already credited with in affections of the mucous membranes of the throat and stomach.

With these very favorable experiences on record the failures recorded by Nogues were so much more surprising. In three cases of stone, five tuberculous and four other cystites, he experienced in most instances an increase of the troubles and no cessation of the pain from orthoform therapy. The reason for these failures is to be found in the fact that Nogues used glycerin as the vehicle for the introduction of the orthoform, on account of the solubility of orthoform in glycerin in the proportion of 5 in 100 parts, and instilled this solution two or three times daily, how much, however, is not stated. Now it is a well-known fact that gylcerin applied to excoriations produces violent burning, and that the muscles of the bladder react by contraction from the use of glycerin. For these reasons glycerin is not a fit vehicle for the introduction of orthoform into the bladder, as the glycerin would exert these two very harmful properties before the orthoform would get a chance to act. The orthoform besides would be precipitated as soon as it came in contact with the aqueous urine. A solution of orthoform in glycerin is therefore of no practical value. Besides it is impossible to introduce enough orthoform into the bladder by installation; in using a 5 per cent. glycerin solution it would be necessaiy to use 20.0 which would be impracticable. A physiological sodium chlorid solution (1·to 30.0 or 50.0) that neither irritates the muscles of the bladder nor the ulcers, is, therefore, preferred as a means of irrigation. This solution can be eminently recommended for use in allaying pain of sufferers from bladder troubles whose general condition is usually very much disturbed.

The possibility of the formation of stone might be advanced, as a theory, by the presence of the almost insoluble orthoform in the bladder. This does not seem probable, however, as no such symptoms have been known in many cases treated. Also as acid as well as alkaline urine will dissolve small quantities of the orthoform, the greater part would soon be removed by frequent micturitions and irrigation of the bladder, besides new solvent fluids being constantly thrown off from the kidneys, the possibilities of stone are very remote. There are furthermore few cases in which a lasting use of orthoform is indicated, except in the case of malignant growths, etc. Mirabeau had good results from orthoform suspended in boric acid solution in anesthetising the bladder and reducing the tension so that it was possible to use the cystoscope upon very sensitive bladders without pain. The good results obtained in the large number of cases (330) treated within two years with orthoform, teach us that the prime object of its use—anesthesia of wounds—was realised in all but a very few exceptional cases. Further, the circumstance that in the many cases of continued use of orthoform, some for weeks and months at a time, no serious after-effects occurred, is the surest evidence of the harmlessness of orthoform when correctly used.

Only a few local disturbances could be traced to its use. These were mostly vesicular or pustular eczemas, that appeared with moderate itching in the neighborhood of the wound. Of these only 5 in 330 cases occurred. Orthoform gangrene is still rarer. The prime condition essential to the appearance of orthoform eczema seems to be individual intolerance (idiosyncrasy). Too copious use of the powder and localisation of the affection treated are also considered factors in developing eczema. The appearance of orthoform necrosis would necessitate as prime factors, besides individual idosyncrasy, particularly malnutrition of the tissues under the unfavorable influence of an infection or hyperemia. Keeping these conditions in mind it is easy to avoid the occurrence of these after-effects. Then orthoform should only be used under the direct supervision of a physician; self-application by the patient should not be permitted, as the patient is liable to be deceived as to his condition. The physician can prevent the eczema by avoiding the use of orthoform too freely in the beginning, and by the application of a thick zinc oxide paste around the ulcer. If in spite of this precaution eczema should appear, a discontinuance of the orthoform and applications of a powder and bandaging for two or three days will remedy the trouble. To avoid gangrene, especially in persons afflicted with venous hyperemia of the lower limbs, the patient should be kept strictly in bed, and where infection of the ulcers is suspected treatment with the usual antiseptcs must not be neglected. The experiments of the author have further proven that orthoform does not increase the secretions or retard the process of healing.

More we need not wish for. It suffices that orthoform, used in reasonable quantities, especially under the direction of a physician, is an absolutely harmless local anesthetic with which the surgeon is capable of banishing the pain of wounds with an astounding degree of certainty.

---

In order to relieve the pain and irritation caused by the removal of dressings adhering to a wound, pour some peroxide of hydrogen over the adherent part of the dressing. This will rapidly soften the coagulated discharges and the dressing will come off readily. This method saves the time employed in prolonged soaking with ordinary solutions, and relieves the apprehension so usually shown by patients at each fresh dressing.—*Canadian Practitioner and Review.*

